# Task relevance modulates the behavioural and neural effects of sensory predictions

**DOI:** 10.1371/journal.pbio.2003143

**Published:** 2017-12-04

**Authors:** Ryszard Auksztulewicz, Karl J. Friston, Anna C. Nobre

**Affiliations:** 1 Oxford Centre for Human Brain Activity, Department of Psychiatry, University of Oxford, Oxford, United Kingdom; 2 Department of Biomedical Sciences, City University of Hong Kong, Hong Kong; 3 Wellcome Trust Centre for Neuroimaging, Institute of Neurology, University College London, London, United Kingdom; University of Birmingham, United Kingdom of Great Britain and Northern Ireland

## Abstract

The brain is thought to generate internal predictions to optimize behaviour. However, it is unclear whether predictions signalling is an automatic brain function or depends on task demands. Here, we manipulated the spatial/temporal predictability of visual targets, and the relevance of spatial/temporal information provided by auditory cues. We used magnetoencephalography (MEG) to measure participants’ brain activity during task performance. Task relevance modulated the influence of predictions on behaviour: spatial/temporal predictability improved spatial/temporal discrimination accuracy, but not vice versa. To explain these effects, we used behavioural responses to estimate subjective predictions under an ideal-observer model. Model-based time-series of predictions and prediction errors (PEs) were associated with dissociable neural responses: predictions correlated with cue-induced beta-band activity in auditory regions and alpha-band activity in visual regions, while stimulus-bound PEs correlated with gamma-band activity in posterior regions. Crucially, task relevance modulated these spectral correlates, suggesting that current goals influence PE and prediction signalling.

## Author summary

As natural environments change, animals need to continuously learn and update predictions about their current context to optimize behaviour. According to predictive coding, a general principle of brain function is the propagation of both neural predictions from hierarchically higher to lower brain regions and of the ensuing prediction-errors back up the cortical hierarchy. We show that the neural activity that signals internal predictions and prediction-errors depends on the current task or goals. We applied magnetoencephalography and computational modelling of behavioural data to a study in which human participants could generate spatial and temporal predictions about upcoming stimuli, while performing spatial or temporal tasks. We found that current context (task relevance) modulated the influence of predictions on behavioural and neural responses. At the level of behavioural responses, only the task-relevant predictions led to improvement in task performance. At the level of neural responses, we found that predictions and prediction-errors correlated with activity in different brain regions and in dissociable frequency bands—reflecting synchronized neural activity. Crucially, these specific neural signatures of prediction and prediction-error signalling were strongly modulated by their contextual relevance. Thus, our results show that current goals influence prediction and prediction-error signalling in the brain.

## Introduction

The notion that the brain generates internal predictions to optimize behaviour is now well established [1–3]. Within the predictive-coding framework, predictions ground perceptual inference and are thought to be conveyed by descending connections in cortical hierarchies [4,5], which may be mediated by synchronized activity in the alpha- and beta-bands [6]. Conversely, incoming sensory or neural inputs—that are unexplained by predictions—translate into sensory prediction error (PE) signals. These “newsworthy” signals induce neural responses [4], which are thought to be propagated up sensory hierarchies in higher frequency bands such as gamma [5,6]. Accordingly, the modulation of alpha- and beta-band activity due to anticipatory predictions has been demonstrated in several modalities (visual: [7,8], auditory: [9,10], somatosensory: [11], motor: [12], see also [13]). Similarly, gamma-band PE signalling has been shown in visual [14] and auditory cortices [9,15].

Predictions can be generated about multiple attributes of stimuli, including their constituent features and their location and time of onset. Indeed, spatial and temporal predictions have been shown to act synergistically to improve visual discrimination in cued orienting tasks [3,16,17,18,19]. However, in natural cases, predictions are typically not cued but evolve dynamically (i.e., predicting the implications of hearing a car’s horn depends on the current context [e.g. location, traffic conditions, driving culture]). While some previous studies have shown that stimulus predictions can be generated and employed even when they are not behaviourally relevant [20–22], other findings suggest that the difference in neural activity triggered by predicted versus unpredicted targets is amplified by attention [23], and predictions about upcoming targets are learned and exploited more efficiently than predictions about nontargets [24]. However, it is not known whether predictions of multiple stimulus attributes are learned independently, or if the task relevance of specific predictions modulates their encoding and updating. Thus, while predictability and task relevance could constitute 2 independent sources of top-down control [25], relevance could also affect the deployment of predictions, precluding redundant or wasteful processing of task-irrelevant sensory information. In other words, predictability and task relevance may interact in selecting the most informative and relevant PEs for belief updating.

Here, to test whether the effect of predictability depends upon task relevance, we designed a task in which participants could use fluctuating spatial and temporal predictions to report either the location (left/right hemifield) or the latency (early/late relative to the cue) of visual targets. Predictions guiding task responses could be formed at different hierarchical levels of processing; at the lower level, participants could use a cue predicting the location/latency of the target in a given trial. At the higher level, they could learn the validity of the cue over several trials. We inferred the participants’ trial-by-trial predictions and PEs using an ideal-observer model based upon a hierarchical Bayesian inference [26–31]. The resulting predictions and PEs, as well as their interactions with task relevance, were used to explain time-frequency (TF) responses (measured with magnetoencephalography [MEG]) to test whether the neural correlates of predictions and PEs are modulated by task relevance.

## Results

### Behavioural results

**Fig 1:**
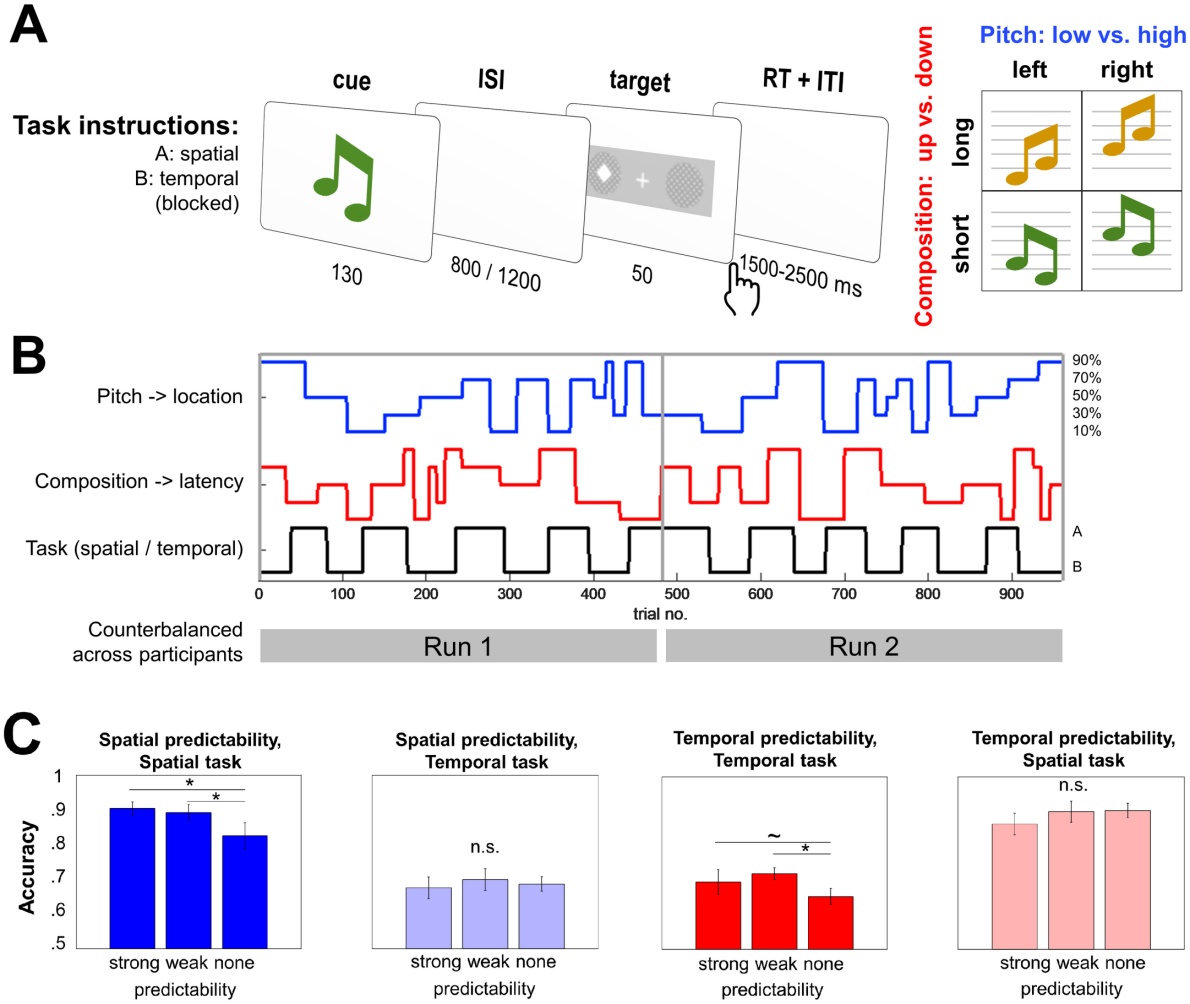
Task design and performance. (A) Participants performed location and latency discrimination of visual targets. An auditory cue consisted of an ascending (orange) or a descending (blue) pair of tones (cue composition), presented at a high or low pitch. The pitch of the cue could predict the location (left versus right) of the visual target, while the composition of the cue could predict the latency (early versus late) of the target, followed by a speeded discrimination response. Participants performed location or latency discrimination in separate blocks. (B) Cue validity varied unbeknownst to the participants over the course of the experiment. Spatial (blue) and temporal (red) validity levels were uncorrelated and changed implicitly. Alternating tasks (black) were prompted by explicit instructions. (C) Predictability interacted with task relevance in both tasks, improving accuracy when the predictions were relevant. The main effect of relevance reflected the differences in accuracy between tasks. *N* = 17; error bars: SEM; post-hoc *t* tests * *p* < 0.05; ^~^ *p* < 0.1. Data pertaining to this figure are available on Figshare https://figshare.com/s/2d2755bfdeea1cbb415f. ISI, inter-stimulus interval; ITI, inter-trial interval; n.s., not significant; RT, reaction time.

Participants performed 2 tasks—location and latency discrimination of visual targets—in alternating blocks (Fig 1A). Each trial contained an auditory cue (a tone pair) and a visual target (a near-threshold square embedded in noise and presented peripherally). The auditory cue had the following 2 features: pitch (high versus low) and composition (ascending or descending pair). Similarly, the visual target had the following 2 features: location (right versus left hemifield) and latency (approximately 730 or approximately 1,270 ms after the cue). The following 2 cue-target contingencies were introduced in the task: cue pitch could predict target location and cue composition could predict target latency, with a varying degree of validity (Fig 1B). Participants were not informed of the cue-target contingencies or cue-validity manipulations. Thus, in certain (predictive) trials during each task block, the cue could be used to implicitly predict target location and/or latency, whereas in other trials, the cue was uninformative with respect to 1 or both target features. However, the task relevance manipulation was introduced explicitly, i.e., at the beginning of each block participants were informed whether they should discriminate the location or the latency of the target. Because cue validity varied along spatial and/or temporal dimensions, this design enabled us to orthogonalize predictability and task relevance, i.e., a stimulus could be predictable or not in the relevant or irrelevant context (determined by the current task).

In both tasks, cue validity led to improvements in discrimination accuracy depending on task relevance (Fig 1C). Thus, the interaction between predictability (a parametric factor encoding 90, 70, or 50% cue validity) and relevance (e.g., relevant: spatial predictability in a spatial task; irrelevant: spatial predictability in a temporal task) was significant for both tasks (spatial: F_1,50_ = 5.23, partial η^2^ = 0.09, *p* = 0.02; temporal: F_1,50_ = 5.10, partial η^2^ = 0.09, *p* = 0.02). In both tasks, the main parametric effect of predictability was not significant (*p* > 0.05, F < 2). However, there was a significant main effect of relevance (spatial: F_1,50_ = 34.61, partial η^2^ = 0.46, *p* < 0.001; temporal: F_1,50_ = 7.03, partial η^2^ = 0.15, *p* = 0.01), reflecting better overall performance in the spatial task (spatial: mean 87.2%, SEM 2.2%; temporal: mean 68.7%, SEM 2.2%) in the analyzed trials. No effect of the foreperiod on accuracy was observed in either task (paired *t* tests: short versus long intervals; both *p*’s > 0.2).

### Behavioural modelling

**Fig 2:**
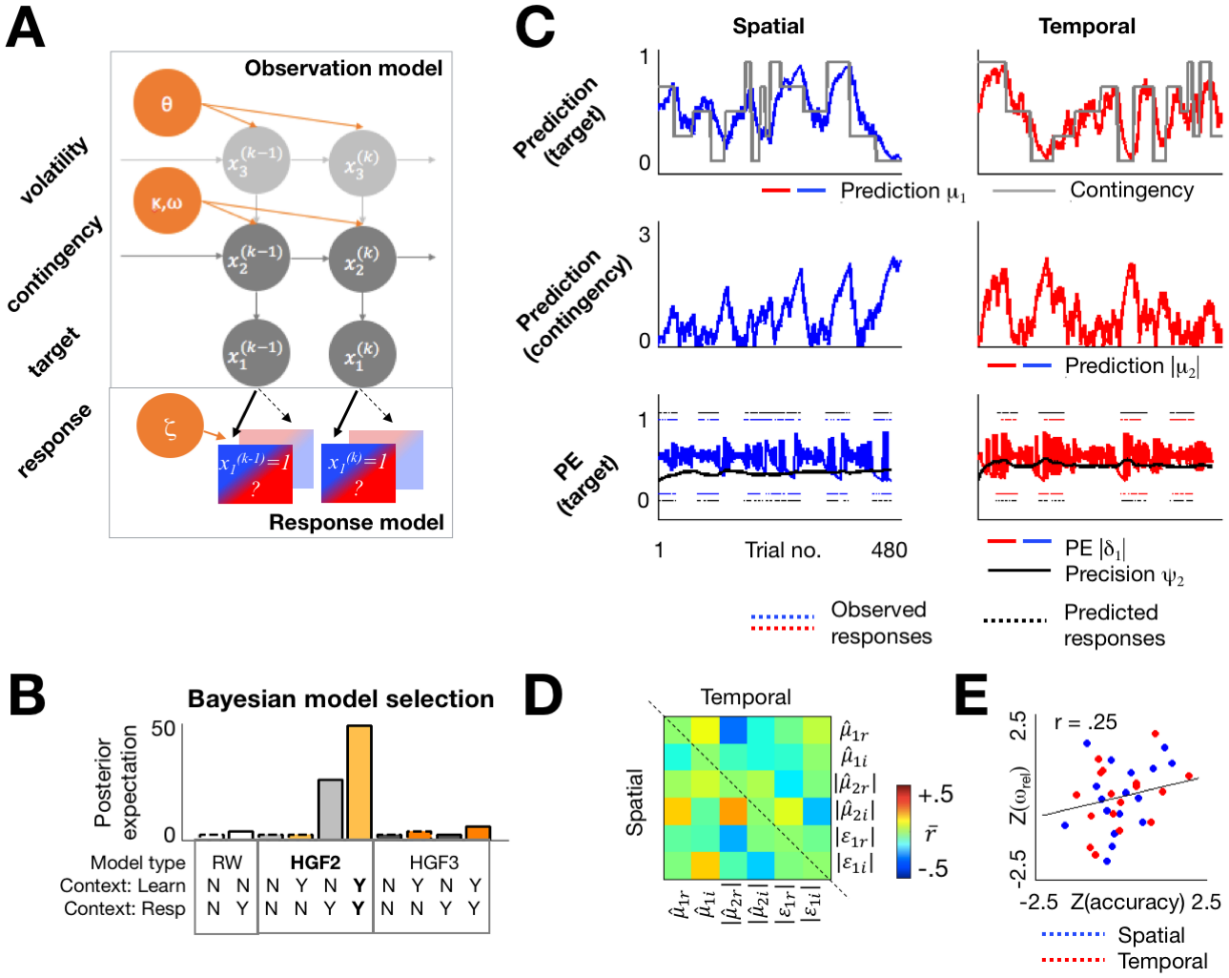
Behavioural modelling. (A) The HGF comprises an observer part, describing the beliefs inferred at 3 levels (low: predictions about target location/latency; middle: cue-target validity level; high: volatility of cue validity), and the response part, linking these beliefs to predicted responses. The full model assumes all 3 levels and a weighted influence of relevant (saturated blue/red) and irrelevant (unsaturated) predictions on participants’ responses. Grey: model states; orange: model parameters. (B) Three alternative observer models (HGF3, HGF2, RW) and 2 alternative response models (task-general: weighted influence of relevant and irrelevant predictions; task-specific: exclusive influence of relevant predictions) were subject to Bayesian model selection. Plot shows log-model evidence relative to the weakest model and indicates task-specific HGF2 as winning. (C) HGF-derived trial-by-trial time-series (representative participant) of predictions about target location/latency (${\hat{\mu }}_{1}$; upper panels) and cue validity (${\hat{|\mu }}_{2}|$; middle panels) and PEs about target location/latency (|*ε*_2_|; lower panels). (D) Mean correlations between HGF regressors. (E) Correlation between the prior variance of validity level updates and mean accuracy across participants. Data pertaining this figure are available on Figshare https://figshare.com/s/2d2755bfdeea1cbb415f. HGF, Hierarchical Gaussian Filter; HGF2, 2-level HGF; HGF3, 3-level HGF; PE, prediction error; RW, Rescorla-Wagner.

To explain the interaction between predictability and relevance on accuracy, we modelled individual participants’ responses using a Hierarchical Gaussian Filter (HGF) [26]. This Bayesian observer model allowed us to infer, on a trial-by-trial basis, the participants’ beliefs in terms of predictions and PEs about targets and cue validity levels. The HGF comprises an observer model, describing how the participants’ beliefs about various hierarchical aspects of the task are updated given trial outcome, and a response model, linking these beliefs to behavioural responses (Fig 2A). The observer model assumes that participants can form beliefs about 3 hierarchical aspects of uncertainty entailed by the task: (1) target location and/or latency in a particular trial (given the cue), (2) the current cue validity level, and (3) the current volatility (i.e., how fast cue validity changes over trials). Because task relevance was introduced in a deterministic (rather than probabilistic) way, we modelled relevance as a set of weights quantifying the contribution of predictions to the response in a given trial. By fitting the model to behavioural data, one can estimate the evidence for a particular model (quantifying how well the model explains the data, while penalizing for model complexity) and the model parameters. These parameters describe individual differences in learning and trial-by-trial expectations that generate predictions and PEs at various hierarchical levels.

We compared 5 alternative observation models: first, we specified 3-level HGFs (HGF3; in which participants’ beliefs at all levels are updated and can influence behaviour) in which the learning parameters could be either context-specific (i.e., the contribution of PEs to prediction updates could vary between task-relevant and task-irrelevant context) or nonspecific; in the same way, we specified 2-level HGFs (HGF2; in which changes in volatility are not inferred), again with context-specific or nonspecific learning parameters; finally, for a comparison with the Bayesian models we added a standard reinforcement-learning model (with a fixed learning rate). We also specified 2 alternative response models: task-specific (in which spatial/temporal predictions are used only to model responses in the spatial/temporal task) and task-general (in which both types of predictions contribute to responses in both tasks). Thus, our model space contained 10 models.

A random-effects Bayesian model comparison revealed that the winning model was HGF2, with context-specific learning parameters and observation parameters (Fig 2B; protected exceedance probability >95%, Bayesian omnibus risk *p* < 0.001, indicating very strong evidence for the winning model; cf. [32]). This suggests that our participants did not infer changes in volatility [33] and that their beliefs about target outcomes influenced learning and behaviour in task-relevant contexts only. The prior and posterior model parameters are provided in the Materials and methods section. Across tasks, the posterior learning parameter ω_rel_ of the observation model (denoting the weight of context-relevant PEs in updating subsequent predictions; see Eqs 14 and 15 in Materials and methods) was the only significant predictor of individual participants’ mean accuracy out of the 4 free model parameters considered (stepwise regression: β = 0.25, *p* = 0.04; see Materials and methods and Fig 2E). This between-subject correlation provides an important validation of the within-subject model of behaviour, and suggests that the degree to which individuals learn predictions in task-relevant contexts is relevant for adaptive behaviour. Furthermore, the learning parameters ω_rel_ and ω_irrel_ were significantly different within participants (repeated-measures ANOVA with factors Relevance and Task; Relevance: F_1,16_ = 5.11, partial η^2^ = 0.15, *p* = 0.037; Task and Interaction: *p* > .25), providing further evidence that the learning parameters were sensitive to the observed behavioural effects of contextual relevance.

Example time-series of predictions and PEs (from one participant) are shown in Fig 2C. Beliefs about the most likely location and latency in a given trial (${\hat{\mu }}_{1}$) track the evolving contingencies (pitch–location and composition–latency), suggesting that participants learn the objectively defined cue-validity level (although it is not an input to the model). Accordingly, predictions about cue validity ${\hat{\mu }}_{2}$ are highest for strongly predictable trials. Furthermore, PEs about target locations/latencies (δ_1_) increase when the outcome in a given trial does not match the prediction, and gradually decrease as the participant learns a new validity level and the respective precision ψ_2_ ramps up. This precision term is in turn used to weight the influence of the PEs on prediction updates. To relate predictions and PEs to neural activity, we used (unsigned) precision-weighted PEs ε_i_ = ψ_i_δ_i-1_ (see Eq 8), in addition to predictions about cue validity ${\hat{\mu }}_{2}$, as regressors to explain TF power of the MEG responses. The mean correlation between the regressors did not exceed r = 0.25, consistent with previous studies using the HGF ([27,29]; Fig 2D), and warranting their use as independent regressors in the analysis of neural activity.

### TF responses

**Fig 3:**
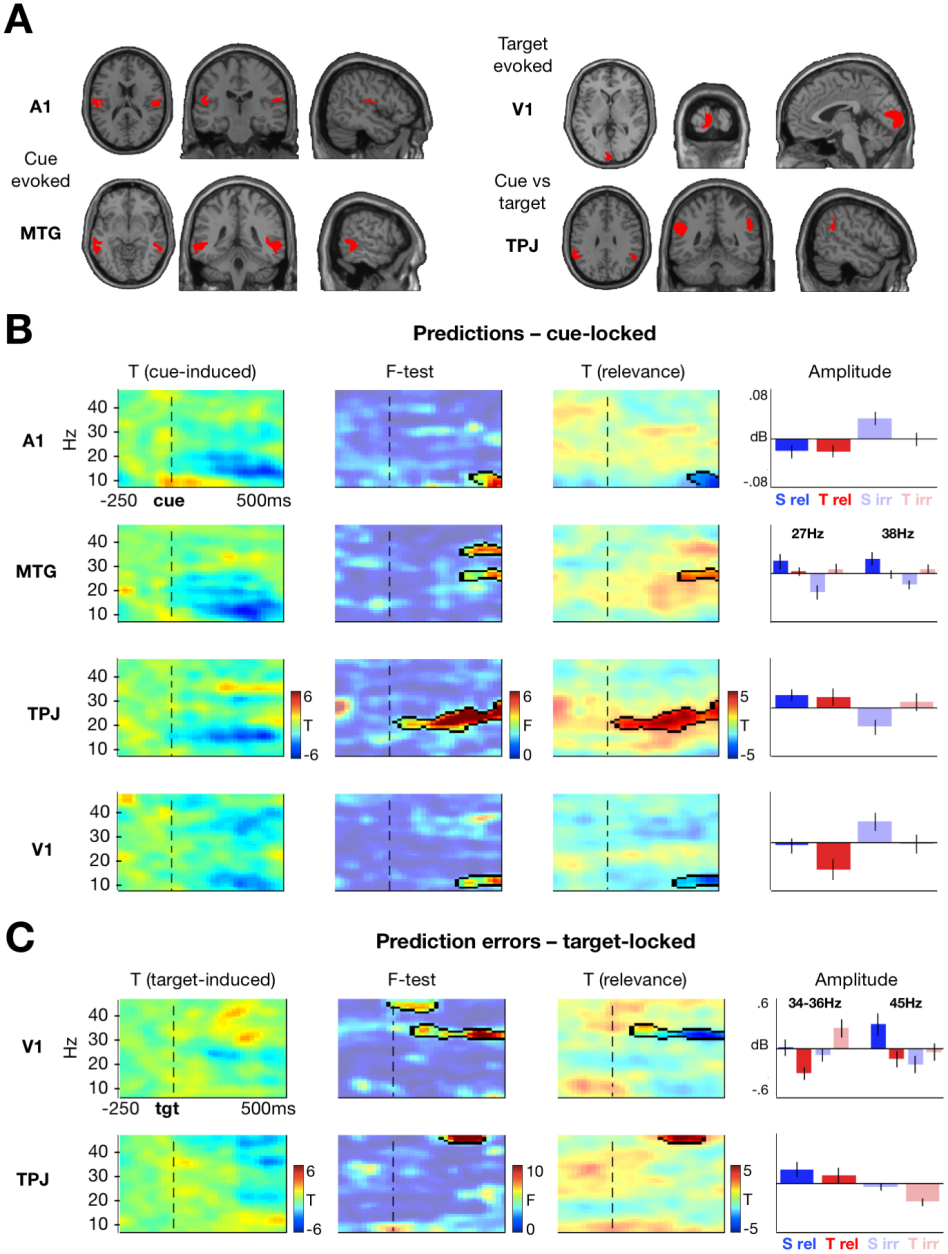
Neural correlates of predictions and PEs. (A) Source reconstruction. Auditory cortex (slices centred at MNI 50, −22, 18) and MTG (MNI 62, −40, −8) were identified as main sources of cue-evoked activity. TPJ (MNI 52, −48, 30) differentiated between cue- and target-induced responses. V1 (MNI −2, −100, 2) was the main source of target-evoked activity. (B) Cue-induced prediction correlates are modulated by task relevance. Plots show TF maps of (far left) cue-induced activity independent of any modulation by prediction type or relevance; (mid left) F-contrast across 4 conditions (spatial relevant; spatial irrelevant; temporal relevant; temporal irrelevant), indicating clusters of activity showing significant differences between the conditions; (mid right) T-statistic map of the main effect of relevance, indicating significant differences between relevant versus irrelevant predictions; (far right) parameter estimates per condition for the significant cluster (error bars: SEM). Dashed line marks cue onset. Outlines show F-contrast clusters significant at *p*_FWE_ < 0.05. (C) Target-induced PE correlates are modulated by task relevance. Plots show TF maps of (far left) target-induced activity independent of any modulation by PE type or relevance; (mid right) T-statistic map of the main effect of relevance, indicating significant differences between relevant versus irrelevant PEs. Dashed line marks target onset. Cluster outlines, mid-left and far-right panels as in (B). Data pertaining this figure are available on Figshare https://figshare.com/s/2d2755bfdeea1cbb415f. A1, primary auditory cortex; MNI, Montreal Neurological Institute; MTG, middle temporal gyrus; PE, prediction error; TF, time-frequency; TPJ, temporoparietal junction; V1, calcarine cortex.

To reduce MEG data dimensionality, analysis was performed in source space after localizing the principal sources involved in cue and target processing (Fig 3A; Table 1). Source reconstruction showed that (auditory) cues evoked activity in bilateral primary auditory cortex (A1) and middle temporal gyrus (MTG), whereas (visual) targets evoked activity in the region of calcarine cortex (V1). Additionally, cues induced more activity in the bilateral temporoparietal junction (TPJ) than targets. Source-level time-series were extracted from each source and transformed into TF estimates for the entire experimental session, averaging across hemispheres to avoid a multiple comparisons problem across unilateral regions in analysing the TF responses and in subsequent modelling. Thus, the main analysis focused on the modulation of the neural correlates of prediction and PE signalling independent of their possible lateralization. Participant-specific model-based sequences of predictions and PEs were used as regressors in a convolution general linear model (GLM) of TF responses [34]. The convolution model enabled us to detect significant parametric effects of predictions (|${\hat{\mu }}_{2}$|) and PEs (|ε_2_|) on responses in each region. To test for modulatory effects of contextual relevance on prediction and PE signalling, regressors were entered separately for task-relevant and task-irrelevant contexts (thereby modelling an interaction).

**Table 1:** Source reconstruction results.

Contrast	Region	MNI	T_peak_	Voxel Extent	*p*_FWE_ (Cluster)
Cue versus baseline	R MTG	44, −34, 4	7.68	584	< 0.001
	L MTG	−50, −48, −2	7.14	936	< 0.001
	R AC/PT	50, −22, 18	5.88	221	< 0.001
	L AC/PT	−50, −28, 16	5.44	389	< 0.001
	R MTG	50, −62, 20	6.68	373	< 0.001
Target versus baseline	L V1	−2, −100, 2	7.22	572	< 0.001
Cue versus target	L SMG/TPJ	−52, −38, 26	7.40	579	< 0.001
	R SMG/TPJ	52, −48, 30	6.98	165	< 0.001
	R AC/PT	48, −22, 18	6.75	28	< 0.001
	L MTG	−56, −38, 0	5.90	15	0.001
	L MTG	−50, −66, 20	5.89	53	< 0.001
	R AC/PT	56, −16, 14	5.62	12	0.002
	R MTG	52, −62, 14	5.57	13	0.001
	L SMG/TPJ	−42, −34, 44	5.33	19	0.001

Statistical parametric maps of source activity estimates were thresholded and corrected at *p*_FWE_ < 0.05 (cluster-level; minimum cluster extent: 10 voxels). AC, auditory cortex; L, left; MTG, middle temporal gyrus; PT: planum temporale; R, right; SMG, supramarginal gyrus; TPJ, temporoparietal junction; V1, calcarine cortex.

As for the behavioural data, neural effects of predictions depended on task relevance. In the analysis of the simple main effects of predictions and PEs (i.e., ignoring their task relevance), no effect survived statistical significance testing (TF clusters thresholded at *p* < 0.05, uncorrected across TF points and Bonferroni-corrected across brain regions). However, testing for the effect of relevance on the TF correlates of predictions and PEs yielded several significant clusters of activity in cue- and target-processing regions (Table 2). Specifically, relevant predictions increased post-cue beta power in MTG and TPJ and decreased post-cue alpha power in A1 and V1 (Fig 3B), while PEs increased post-target gamma power in TPJ and V1 and decreased beta-band power in V1 (Fig 3C).

**Table 2:** Effects of trial-by-trial predictions and PEs on TF responses.

Regressor	Source	TF Cluster	*p*_FWEclust_	F_peak_	Z_peak_	ANOVA Effect	F_1,16_	*p*	t_16_
Predictions (cue-locked)	A1	9 Hz, 430 ms	0.024	9.26	3.84	Rel-Irrel	8.93	0.009	−2.99
	MTG	27 Hz, 310–430 ms	0.047	8.05	3.55	Rel-Irrel	7.54	0.014	2.75
						Interaction	11.51	0.004	3.39
		38 Hz, 370–430 ms	0.039	8.21	3.59	Interaction	11.13	0.004	3.34
	TPJ	21–27 Hz, 90–450 ms	< 0.001	16.38	5.09	Rel-Irrel	5.27	0.035	2.29
	V1	11–12 Hz, 330–430 ms	0.01	8.29	4.12	Rel-Irrel	4.96	0.04	−2.24
						Spatial-Temporal	6.4	0.022	2.52
PEs (target-locked)	TPJ	48 Hz, 270 ms	0.023	17.65	5.26	Rel-Irrel	10.95	0.004	3.31
	V1	34–36 Hz, 130–350 ms	< 0.001	12.40	4.47	Rel-Irrel	4.99	0.04	−2.23
						Interaction	12.77	0.002	3.57
		45 Hz, 90 ms	0.028	7.31	3.36	Interaction	14.84	0.001	3.85

Interaction effects correspond to a contrast of spatial (relevant > irrelevant) > temporal (irrelevant > relevant). A1, primary auditory cortex; MTG, middle temporal gyrus; PE, prediction error; Rel-Irrel, relevant-irrelevant; TF, time-frequency; TPJ, temporoparietal junction; V1, calcarine cortex.

Beyond the modulation of prediction and PE signalling by relevance, cue-induced alpha-band responses in V1 differentiated between the spatial and temporal prediction estimates (Table 2 and Fig 3B). Furthermore, beta-band prediction signalling in MTG and PE signalling in V1 were modulated by task relevance and cue-target contingency, such that spatial predictions showed a stronger modulation by relevance than temporal predictions in MTG, whereas spatial PEs showed a weaker modulation by relevance than temporal PEs in V1 (Table 1 and Fig 3B and 3C). The latter finding might reflect a lateralization effect, whereby PE signalling in the temporal task is likely nonlateralized and as a result its modulation might be easier to detect in source activity averaged across hemispheres. Thus, we performed an additional control analysis to assess whether PE signalling is indeed more lateralized in the spatial task. To this end, we re-ran convolution modelling using a signed PE regressor, as opposed to the unsigned PE regressor used in the main analysis; thus, the signed PE regressors had positive values for unexpected targets on the left (or at early latencies), and negative values for unexpected targets on the right (or at late latencies). We reasoned that by using the signed PE regressor, in the spatial task source-level activity linked to PE signalling in different hemispheres in V1 should have the opposite sign (due to hemifield-specific PE signalling), whereas in the temporal task they should have the same sign (because PEs regarding target latency will be processed in both hemifields to a similar extent). We used a region-of-interest approach, focusing on the significant clusters identified in the main analysis (Fig 3C), whereby the mean spectral power was extracted from these clusters per hemisphere (left/right), task (spatial/temporal), context (relevant/irrelevant), and participant, and entered into an ANOVA with 3 factors (hemisphere, task, and context). As hypothesised, we did observe a significant hemisphere effect for spatial PEs in V1 (F_1,64_ = 8.29, *p* = 0.01), in addition to a main effect of relevance (F_1,64_ = 4.74, *p* = 0.04). In the temporal task, however, the main effect of hemisphere was not significant (F_1,64_ = 0.55, *p* = 0.46), although the effect of relevance was preserved (F_1,64_ = 4.33, *p* = 0.04). The remaining main or interaction effects were not significant.

To control for the possibility that the effects of predictions on neural responses might be contaminated by a differential processing of specific auditory cues (e.g., either their pitch or composition being more salient and therefore easier to process), we ran an additional control analysis testing whether source-level activity showed differential effects of pitch and/or composition. To this end, we repeated the analysis of cue-induced (i.e., prediction-related) responses with additional regressors coding for cue pitch (high versus low) and composition (ascending versus descending). Our rationale was that, in addition to treating pitch and composition as possible confounding factors on their own, any difference in variance explained by the 2 features respectively would be accounted for by these confound regressors and effectively removed from prediction-related activity. The inclusion of these regressors did not change the results in TF space: all the significant clusters identified before showed the same patterns of condition-specific differences as reported in Fig 3B, using identical significance criteria as in the original analysis (i.e., correcting for multiple comparisons across regions using Bonferroni correction, and for TF points using family-wise error ratio under random field theory assumptions).

### Effective connectivity

To test the directionality of the effects identified above, we used dynamic causal modelling (DCM) for TF responses [35]. This phenomenological Bayesian modelling approach allows a quantification of the effective (directional) connectivity between different regions and frequency bands. Within- and cross-frequency amplitude–amplitude coupling was analysed in 2 time windows: 0–500 ms relative to the cue onset (in which TF activity was found to be modulated by prediction relevance; Fig 3B), and 0–500 ms relative to the target onset (in which TF activity was modulated by PE relevance; Fig 3C). In the analysis of the cue-processing period, we modelled connectivity in a network of 4 sources sensitive to prediction relevance: A1, MTG, TPJ, and V1. Similarly, when analysing target-induced activity, effective connectivity was modelled in a network of 2 sources: V1 and TPJ.

**Fig 4:**
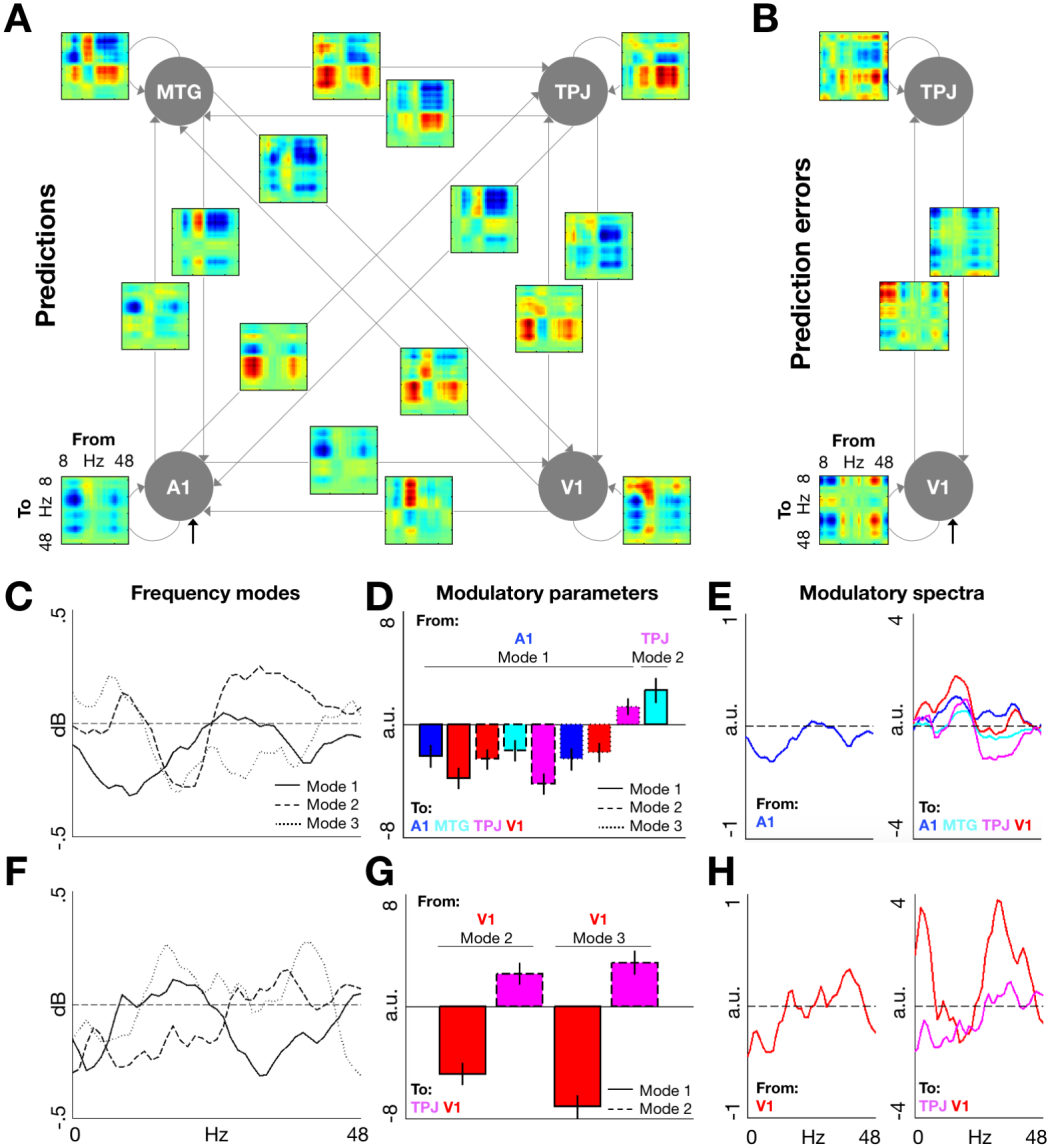
DCM. (A) Frequency-by-frequency maps of modulatory effects of contextual relevance on prediction processing. Effects were modelled in a network of 4 interconnected areas, corresponding to the 4 regions in which significant effects of relevance on prediction-related responses were identified (cf. Fig 3B). (B) The corresponding maps of modulatory effects of contextual relevance on PE processing, modelled in a network of 2 areas in which significant effects were identified (cf. Fig 3C). (C) Principal frequency modes estimated for prediction-related responses across the modelled areas. (D) Significant modulatory parameters corresponding to the effects of contextual relevance on prediction-related responses. Each bar represents a significant modulation (by contextual relevance) of the influence of a particular frequency mode in 1 region on another frequency mode in another region. (E) Modulatory spectra of the relevance-related effects of A1 activity (left panel, corresponding to Mode 1) on prediction-induced activity in all regions (right panel, corresponding to an average across frequency modes weighted by the respective modulatory parameters). (F-H) Same as (C-E) but for PE processing (H, left panel: an average of Modes 2 and 3). Data pertaining this figure are available on Figshare https://figshare.com/s/2d2755bfdeea1cbb415f. A1, primary auditory cortex; DCM, dynamic causal modelling; MTG, middle temporal gyrus; PE, prediction error; TPJ, temporoparietal junction; V1, calcarine cortex.

Fig 4 provides frequency-frequency coupling maps mediating (Fig 4A and 4B) the modulation of prediction and PE signalling by relevance, and (Fig 4C–4H) the significant modulatory parameter estimates quantifying the effects of relevance on cross-frequency coupling within and between regions. Regions involved in prediction (Fig 4A) and PE (Fig 4B) signalling showed the spectral asymmetry between ascending and descending connections, as suggested previously [5]. Specifically, in prediction signalling, ascending connections from A1 to TPJ, from V1 to MTG, and from V1 to TPJ showed strong excitatory effects in higher frequency ranges, whereas the respective descending connections showed net inhibitory effects in low-frequency ranges (Fig 4A). Similarly, in PE signalling, the ascending connection from V1 to TPJ mediated primarily excitatory effects (in both high and low frequency bands), whereas the descending connection mediated primarily inhibitory effects [5]. Upon closer inspection of the significant modulatory parameters of contextual relevance on prediction processing (Fig 4C–4E), task relevance primarily modulated the influence of low-frequency (alpha-beta) activity in A1 on low-frequency activity throughout the network, having a negative net effect on alpha-beta power in all regions. Additionally, contextual relevance modulated the influence of TPJ on MTG activity (frequency mode 1; Fig 4C), reflecting a further inhibition of alpha-beta activity in MTG. In contrast, PE relevance (Fig 4F–4H) primarily modulated the influence of V1 activity on the network, leading to a shift from lower to higher frequencies in V1, as well as to increased propagation of both high- and low-frequency activity to TPJ. Taken together, our DCM findings expand the previous results on low-frequency prediction signalling and high-frequency PE signalling by characterising the network-wide effective connectivity mediating these spectrally distinct effects.

## Discussion

The present study used a model-based MEG approach, assuming an ideal-observer model of behavioural data acquired in a task that orthogonally manipulated stimulus predictability and relevance. By fitting an HGF model [26] to each participant’s behaviour, we estimated the trial-by-trial predictions and PEs that best explained their performance. These estimates of subjective beliefs were then used as regressors in the analysis of the power of MEG responses. Crucially, we demonstrate an interaction between stimulus predictability and task relevance at the following 2 levels: task performance (accuracy) and neural activity. These converging results suggest that prediction and PE signalling are contextualized by current task goals.

### Predictability improves accuracy but only in relevant contexts

The effects on accuracy extend previous findings, suggesting that stimulus predictability improves performance only when predictions pertain to task-relevant targets [24,36,37]. Accordingly, the validity of cues predictive of the target location (latency) improved accuracy in the spatial (temporal) task (Fig 1C), but not vice versa. The effects of irrelevant predictions were either abolished (in the spatial task; see [23], in which sensory predictions failed to affect processing of irrelevant stimuli) or nominally (but not significantly) reversed (in the temporal task) [38]. Finally, despite prior stimulus titration to 70% accuracy in both tasks, we observed differences in performance between the tasks, most likely due to an asymmetry between spatial and temporal discrimination; namely, successful temporal discrimination was necessarily associated with successful spatial discrimination, but inferring target location did not depend on inferring its latency. Nevertheless, the interaction of relevance and predictability was significant in both tasks and had similar effect sizes, suggesting robustness with respect to performance levels. Taken together, although previous studies suggest that the validity of task-irrelevant cues can be learned [20–22,39; but see 40] and spatiotemporal cues can work synergistically [3,16,17,18,19], we show that the effects of predictable cues on accuracy are strongly modulated by task set.

To explain this context sensitivity, we modelled behavioural data using HGF—an ideal Bayesian observer model of learning under uncertainty—allowing us to reconstruct subjective beliefs about experimental contingencies [26]. Hierarchical Bayesian models, such as the HGF, have been proven powerful in explaining behaviour in volatile and probabilistic tasks, by quantifying trial-by-trial inference. In previous work, the HGF has been applied to probabilistic attentional cueing paradigms [28,29] and used to delineate the functional anatomy [27] and neuromodulatory mechanisms [31] of encoding uncertainty at different hierarchical levels. In our task, uncertainty pertained to (1) target location/latency in a given trial, (2) cue validity level governing several trials, and (3) its volatility over multiple trials. In previous applications of HGF to cueing tasks (with a single cue-target contingency), the HGF3 (under which participants’ estimates at all levels influence behaviour) has typically been selected by Bayesian model comparison [27–30]. In our study, however, a comparison of several alternative observation models indicated a reduced HGF2 was the winning model, suggesting that performance of our participants was Bayes-optimal but not sensitive to changes in volatility [33]. Furthermore, the winning response model allowed only for a task-specific influence of relevant predictions on performance, consistent with the effects on accuracy—and establishing the construct validity of our modelling approach. Interestingly, the winning model implemented predictability and contextual relevance at hierarchically different levels: although predictability (cue validity) corresponds to the hidden state μ_1_ that the model successfully recovers from behaviour (Fig 2C, upper panels), contextual relevance is implemented at the level of weights ζ_rel_ and ζ_irrel_ that link these predictions to the simulated responses. Finally, the winning model included context-specific learning rates (separate for relevant and irrelevant contexts), optimised to each individual’s behavioural performance. At the group level, there was a significant difference between context-relevant and context-irrelevant learning rates. Furthermore, at the between-subject level, the context-relevant learning parameter quantifying the learning rates of spatial predictions in the spatial task, and of temporal predictions in the temporal task, correlated with individual participants’ mean accuracy, providing a further validation of the model.

Our model-based finding suggesting that learning rates depend on contextual relevance might explain the discrepancy between our results and several previous studies that reported the effects of predictability even in task-irrelevant contexts [20–22,39]. In these studies, predictability levels were fixed, unlike in our paradigm, in which cue validity varied over the course of the experiment with participants continuously updating their cue-based predictions of target features. In contrast, our results are fully consistent with previous work suggesting that task relevance facilitates learning of cue-target contingencies [24].

### Relevant predictions and PEs induce distinct neural responses

Further evidence for the interaction between predictability and relevance was seen at the level of neural responses. Here, we used the model-based trial-by-trial estimates of predictions and PEs as regressors in the analysis of MEG responses. This analysis revealed no main effect of predictions or PEs on cue- or target-induced responses. However, there was a significant interaction between task relevance and prediction (following cue onset) or PE estimates (following target onset). Thus, the neuronal responses were in line with the behavioural results and Bayesian model comparison described above, and suggested that the neuronal correlates of predictions and PEs are context sensitive and show an effect of task relevance.

Specifically, relevant predictions were associated with postcue beta-band synchronization and alpha-band desynchronization in auditory regions; most likely involved in the processing of the acoustic cues used in this paradigm. Here, although alpha-band activity was similar across tasks, beta-band modulation was seen predominantly in the spatial task (Fig 3B), possibly reflecting baseline performance differences between the 2 tasks. Both beta-band and alpha-band effects have previously been linked to the processing of predictive auditory stimuli (beta-band synchronization: [41]; alpha-band desynchronization: [42]), and interactive effects of expectation and attention have been identified in auditory beta-band activity [10]. Here, beyond the auditory regions, relevant predictions decreased cue-induced alpha-power in early visual cortex, consistent with previously reported alpha-band modulation due to anticipatory predictions in the visual [7,8,43,44] and other domains [11,12,45]. Although these findings suggest that prediction signalling in low-frequency bands might be a modality-general phenomenon [5], we show for the first time that this effect is modulated by contextual relevance.

Context-sensitive signatures of prediction signalling were seen following auditory cues but were not observed at any fixed latency before target onset, consistent with a recent study showing that the latency of beta-band synchronization is not predictive of the anticipated target latency, but instead locked to the cue onset [45]. Furthermore, prediction signalling was associated with activity in auditory (cue-processing) regions as well as in visual (target-processing) regions, and our DCM-based effective connectivity analysis suggested that the network-wide effects of contextual relevance on prediction processing are predominantly due to the influence of A1 activity on the neural responses throughout the network. Although studies on cross-modal orienting have shown that the effects of cue validity are primarily expressed in target-processing regions [46], previous work on cross-modal expectations suggests that predictions might be generated in 1 modality, but their effects manifest as PEs in another modality [47]. Because PEs are thought to be scaled by expected precision [48,49], our results suggest that predictions might be encoded shortly after the cue onset, but their effects on target processing will entail a modulation of target-induced PE activity.

In contrast to the neural correlates of prediction, relevant precision-weighted PEs were linked to target-induced, gamma-band modulation in posterior regions, which is in line with previous empirical work [9,14,15,50] and theoretical proposals [5]. Specifically, relevant PEs along both spatial and temporal dimensions were associated with increased target-induced, gamma-band responses in the posterior supramodal region TPJ/supramarginal gyrus (SMG), consistent with previous functional MRI (fMRI) correlates of HGF-based PE signalling [27]. Furthermore, relevant PEs decreased high beta-band power (approximately 34–35 Hz, below the range of typical visual gamma MEG responses [5]) in early visual regions, predominantly in the temporal task (Fig 3C) in which PE signalling was not as lateralized as in the spatial task, and thus its modulation easier to detect in source activity averaged across hemispheres (as identified in a control analysis; see TF responses in Results). A boost of gamma oscillations at the expense of lower frequencies [51,52] has often been reported as a correlate of predictability of targets [9,15,50,53,54]. In the effective connectivity analysis, we have identified that the observed modulatory effects of contextual relevance are explained by self-reinforcing alpha-band desynchronisation and gamma-band synchronization in V1. Such spectral shifts of neural responses towards higher frequencies are plausibly explained as a result of modulation of the excitability of principal cells and neuronal time constants that underwrite synaptic gain control [5,55]. The augmented high-frequency responses can then be propagated to higher regions, as suggested by stronger within-frequency coupling between V1 and TPJ identified in our DCM analysis.

The link between precision-weighted PEs and high-frequency responses is consistent with predictive coding, under which stimulus predictability (expected precision) is thought to increase postsynaptic gain of principal cells in superficial layers, typically associated with ascending output in high-frequency bands [5,56]. Previous work trying to disentangle the oscillatory signatures of predictions and PEs yielded evidence converging with our results. In a passive listening paradigm, in which the acoustic stimuli changed according to specific rules, sensory prediction violations (putative PEs) were linked to induced gamma activity, while prediction updates were manifest in the beta-band [9]. Similar results have been reported in an active attentional cueing paradigm, where anticipatory alpha/beta activity scaled with target predictability, while post-target gamma activity increased following sensory mismatch [50]. More generally, the spectral asymmetries between TF activity underlying prediction and PE signalling are consistent with previous postulates that predictions are propagated as descending signals from hierarchically higher to lower regions and mediated by low-frequency (e.g., beta-band) synchronization, while PEs are propagated as ascending signals from lower to higher regions and mediated by high-frequency (e.g., gamma-band) synchronization [5,13]. Here, because increased gamma-band power in the associative TPJ/SMG region preceded decreased lower frequency power in visual regions, our findings raise the possibility that the latter effect reflects a descending prediction update [9] following PE signalling in regions integrating cue and target processing.

Beyond showing spectrally and regionally specific responses corresponding to predictions and PEs, our results indicate that these responses are strongly modulated by their task relevance. Although this context sensitivity of prediction and PE signalling could be explained by an active inhibition of irrelevant features [57], recent evidence suggests that distractor suppression is less flexible than target facilitation [58], making it an unlikely explanation of the effects found in our relatively dynamic task. Alternatively, enhanced signalling of predictions and PEs relevant for the current context might reflect attentional prioritization (increased precision; cf. [48]) of the relevant (i.e., salient, uncertainty reducing) cue features and corresponding target features. This interpretation is consistent with recent evidence suggesting that relevant predictive cues attract gaze ([59]; but see [60] for evidence that stimulus regularity itself does not have to be salient), show enhanced working memory maintenance [61] and are judged more positively by viewers [62]. It is worth noting that, to ensure that the neural signalling of predictions and PEs (and its modulation by task relevance) is not mediated by nonspecific effects of attentional capture such as pupil size [38,63], we treated pupil size as a nuisance regressor in the analysis of TF responses. Thus, the observed neural effects were specifically related to the magnitude of predictions and PEs.

Although the interactive effects of cue validity and task relevance on behavioural and neural responses likely reflect that our participants deployed cue-based predictions to prioritize the contextually relevant visual target features, the same cues could have arguably been used to predict the most likely correct motor response. Although our task was not designed to specifically dissociate cue-target and cue-response mappings, we think that this is an unlikely scenario. First, because in both tasks feedback was given only at a block level and not at a single-trial level, it is unlikely that participants would show an effect of cue validity (interactive with contextual relevance) by learning the cue-response mapping alone. In other words, highly predictive and nonpredictive segments of the experiment could not be differentiated without the participants responding to the visual targets. Thus, our behavioural findings are unlikely to be due to participants dynamically updating their cue-to-response mapping on a trial-by-trial basis. Furthermore, in analysing the neural responses, possible confounds due to motor preparation were controlled for by using convolution modelling instead of more conventional TF analyses of epoched data. Specifically, a parametric regressor coding for which button was pressed in a given trial was included in each participant’s convolution model design matrix, effectively removing the effect of lateralised button press preparation up to 250 ms prior to motor response (i.e., overlapping with the latency of the observed neural correlates of PE processing).

Although several previous studies have used the terms “prediction” (or “expectation”) and “attention” interchangeably, here we followed previous conceptual distinctions [64] in treating expectation as the effect of likelihood of a given stimulus or event on its perceptual and neural processing, and attention as stimulus prioritisation based on its relevance. Although the common interchangeable use of the 2 terms in previous literature can to some extend be attributed to the popularity of classical paradigms (e.g., the Posner paradigm) confounding the 2 factors (i.e., the probability of a stimulus occurring and the probability of the required behavioural report), here we made sure to orthogonalise the probability and behavioural relevance of stimulus features (location and latency). However, besides this well-established distinction, there is a more subtle distinction to be made about expectations of particular stimulus features (e.g., of a particular target occurring on the left and late in a given trial) and the level of its predictability (manipulated here as cue validity). The latter distinction has been discussed more recently in the context of predictive coding [49], in which expectations of specific stimulus contents form first-order predictions, while the degree to which these expectations can be formed form second-order predictions (or predictions of precision). It is worth noting that this dissociation is captured by the HGF, in which both first-order predictions (at the lower level) and second-order predictions (precision ratio at the higher level) can influence behaviour. In the context of predictive coding, first-order predictions are thought to be mediated by descending (inhibitory) connections, silencing the ensuing PEs; second-order predictions, on the other hand, are thought to be mediated by modulatory connections increasing or decreasing the precision (gain) of PEs. As such, however, second-order predictions are akin to attention [16], which has also been linked to precision modulation under predictive coding [48]. At the level of TF responses, increased gain is typically associated with a shift from lower to higher frequency bands [5], as observed in our study. Thus, our findings can be interpreted as reflecting co-modulation of gain by predictability of specific stimulus features and the attentional prioritisation of these features.

### Summary

We show that task relevance of cue and target features modulates performance accuracy, the influence of predictions on behavioural responses (as evidenced by Bayesian modelling), and the neural activity induced by both cues and targets. These findings are in line with the notion that the brain performs hierarchical perceptual inference by comparing sensory inputs with the predictions it generates about its own environment at multiple temporal scales. Crucially, we provide evidence that both the predictions and the ensuing PEs can flexibly adapt to dynamically changing goals.

## Materials and methods

### Ethics statement

This study was approved by the local ethics committee (Inter-divisional Research Ethics Committee, Medical Sciences, University of Oxford, approval ref. no. R48540/RE001) and all investigation has been conducted according to the principles expressed in the Declaration of Helsinki. Written informed consent has been obtained for each participant.

### Participant sample

Healthy volunteers (*N* = 20, 12 females, 8 males; median age 22, range 18–49; all right-handed) were invited to participate in the experiment. All participants had normal hearing, no history of neurological or psychiatric diseases, and normal or corrected-to-normal vision.

### Experimental paradigm

Participants were asked to perform a speeded location (left versus right hemifield) or latency (approximately 0.75 s or approximately 1.25 s relative to an auditory cue) discrimination of visual targets. At the block level, an instruction screen specified which task (location or latency discrimination) should be performed next. Each block consisted of, on average, 48 trials (range 38–58). Participants received feedback about their average accuracy and RT after each block. Each participant completed 20 alternating blocks (10 per task), resulting in 960 trials in total. The duration of the whole experimental session was approximately 1 hour.

All visual stimulation was delivered using a projector (60-Hz refresh rate) in the experimenter room and transmitted to the MEG suite using a system of mirrors onto a screen located approximately 90 cm from the participants. Auditory stimulation was delivered by using MEG-compatible stereo ear tubes.

Each trial started with a display of 2 peripherally located placeholders on either side of a centrally presented fixation cross against a grey background. The placeholders were circles (radius 1.5° of the visual angle) consisting of random white dot patches (30% of the pixels within each circle; refreshed with every screen flip at 60-Hz refresh rate). The circles were located on a horizontal axis, with the centre of each placeholder 4° laterally from the fixation cross. After 500 ms (± 10 ms jitter) of placeholder presentation, an auditory cue was played. The cue consisted of 2 short (66 ms) gapless tone pips with carrier frequencies drawn from 4 possible values (400, 500, 800, and 1000 Hz). The cue was administered in a 2 x 2 factorial manner (factors: pitch and composition), and could therefore consist of pips that were either high (800 and 1000 Hz) or low (400 and 500 Hz) in pitch, forming either an ascending (400–500 Hz; 800–1000 Hz) or a descending pair (500–400 Hz; 1000–800Hz). After a variable delay, the cue was followed by a visual target—a white cardinally or diagonally oriented square (side length equals the radius of the placeholder; 50-ms display duration). The target was also administered in a 2 x 2 factorial manner (factors: location and latency), and could therefore be presented either within the left or the right placeholder, and either early (approximately 0.75 s) or late (approximately 1.25 s) relative to cue onset. The orientation of the target was a task-irrelevant feature introduced so that participants could not form a unique target template. The response buttons were counterbalanced across participants. Consecutive trials were separated by a jittered interval (1500–2500 ms).

Unbeknownst to the participants, cue features (pitch and composition) could predict 1 or both target features (location and latency) with varying validity (90, 70, 50, 30, or 10%) forming 2 contingency time-series: cue pitch could predict target location, while cue composition could predict target latency. The 2 contingency time-series were uncorrelated (r < .0001) and based on a predetermined arbitrary association. For instance, 90% spatial cue validity corresponded to 90% right (left) targets following high (low) pitched cues and 10% left (right) targets following high (low) pitched cues; in the 10% validity level, these proportions were reversed. Validity in each contingency time-series changed on average every 32 trials (range 8–54) and the consecutive validity levels varied pseudorandomly with no repetitions. Additionally, over the course of the experiment, validity could change in a more or less volatile way (on average every 16, 32, or 48 trials—with volatility updated after each 5 validity changes). To facilitate subsequent modelling, the validity time-series were precalculated for 2 runs of 480 trials each and fixed for all participants. The order of the 2 runs was counterbalanced across participants. In the behavioural and MEG analyses of both spatial and temporal predictability we a priori collapsed the 2 strongly predictable (90 and 10%) and the weakly predictable (70 and 30%) validity levels (cf. [33]). Thus, the main factors of interest in our experimental design and analysis were: spatial predictability, temporal predictability (each with 3 levels: strongly predictable, weakly predictable, and unpredictable), and task relevance (2 levels: spatial versus temporal task).

Prior to the main experimental session, we ran a short cue training session in which participants discriminated the pitch or composition of the cue until they reached >95% performance. We then trained them on the main experimental tasks (spatial and temporal discrimination of visual targets presented after the auditory cue, with cue validity changing dynamically just as in the main experiment; min. 50 trials per task). During this training, we administered a target stair-casing procedure; in which we adjusted the contrast of the visual targets to approximately 70% performance (1 up, 2 down procedure with an adaptive step size) in the spatial task, and the relative onset of the early versus late targets to approximately 70% performance in the temporal task. As a result, the mean target contrast was 0.28 relative to the placeholder (SD 0.08, range 0.18–0.53), and the mean asynchrony between early and late targets was 541.6 ms (SD 184 ms; mean early and late latencies 729.2 ms and 1270.8 ms postcue respectively; range of early latencies 600–877.6 ms postcue; range of late latencies 1122.4–1400 ms postcue). Prior to analyzing the behavioral and neural effects of stimulus predictability and task relevance, we excluded data from 1 participant who could not maintain central fixation, and 2 further participants whose mean accuracy in either task was below 55% or above 95%.

### Behavioural analysis and modelling

We analysed accuracy in two 3 x 2 repeated-measures ANOVAs, separately for each predictability manipulation (spatial versus temporal), with the main factors predictability (3 parametrically defined levels: strongly predictable, weakly predictable, and unpredictable) and task relevance (2 levels: relevant and irrelevant). The task-relevant trials corresponded to the spatial (temporal) task when analysing spatial (temporal) predictability; the remaining trials were treated as task-irrelevant. Because cue validity changed unbeknownst to the participants, and thus predictability effects could be offset by the initial trials in each validity level in which the previously learned contingency could be used, mean accuracy scores were calculated based on the second half of trials within each run with stable cue validity. Although synergistic effects between spatial and temporal predictability might have contributed to task performance [3,16,17], a 3 x 3 x 2 repeated-measures ANOVA was not conducted due to a low number of trials (<20) in some cells. Furthermore, trials with RTs longer than median +2 SD were discarded from behavioural and neural analyses.

Beyond testing for the behavioural effects of predictability and relevance, we used individual participants’ behavioural data to infer their beliefs about the targets and validity levels on a trial-by-trial level. Specifically, we modeled individual time-series of responses using a HGF (implemented in a Matlab toolbox available as an open source code: http://www.translationalneuromodeling.org/tapas) that models evidence accumulation or learning at multiple levels, and reconstructs an agent’s beliefs about the causes of their sensory inputs [26]. The model uses a variational approximation to an ideal hierarchical Bayesian observer. By fitting the model to behavioural data, one obtains participant-specific parameters of the model (determining the coupling of hierarchical levels, and thus individual learning time-series) and single-trial predictions and precision-weighted PEs at each level of the model hierarchy.

By design, our task introduced uncertainty at 3 levels: (1) where and when the target will appear in a particular trial; (2) which cue-validity level governs the given trial; and (3) how quickly the cue-validity level changes over time. Accordingly, the model estimates the participants’ beliefs at 3 different levels, corresponding to (1) the location x_s1_ and latency x_t1_ of the target, (2) the pitch-location contingency x_s2_ and the composition-latency contingency x_t2_, and (3) the volatility of these contingencies x_s3_ and x_t3_, respectively. These inferred beliefs are hidden states of the observation model, evolving as a Gaussian random walk, with the hidden states at a given level determining the variance of the random walk at the level below:

$$p(x_{s1,t1}|x_{s2,t2})\;=\;{s(x_{s2,t2})}^{x_{s1,t1}}{\left(1-s(x_{s2,t2})\right)}^{1-x_{s1,t1}}\;=\;Bernoulli(x_{s1,t1};s(x_{s2,t2})),$$ (1)

$$p(x_{s2,t2}^{\left(k\right)}|x_{s2,t2}^{\left(k-1\right)},x_{s3,t3}^{\left(k\right)})\;=\;N(x_{s2,t2}^{\left(k\right)};x_{s2,t2}^{\left(k-1\right)},\text{exp}\;\left(\kappa x_{s3,t3}^{\left(k\right)}+\omega \right)),$$ (2)

$$p(x_{s3,t3}^{\left(k\right)}|x_{s3,t3}^{\left(k-1\right)},\vartheta )\;=\;N(x_{s3,t3}^{\left(k\right)};x_{s3,t3}^{\left(k-1\right)},\vartheta ).$$ (3)

At the lowest level (Eq 1), the prediction of the target location x_s1_ (or latency x_t1_) in a particular trial takes possible values {0; 1} arbitrarily defined in contingency space: i.e., left targets following high-pitch cues and right targets following low-pitch cues are defined as 1, while the opposite locations are defined as 0; similarly, early targets following ascending cues and late targets following descending cues are defined as 1, while the opposite latencies are defined as 0. These low-level predictions are described as a logistic sigmoid function of the respective inferred contingency between the cue and the target x_s2_ (x_t2_), such that if the inferred contingency x_s2,t2_ = 0, both targets (x_s1,t1_ = 1 and x_s1,t1_ = 0) are equiprobable. At the middle level (Eq 2), the inferred cue validity level in a given trial $x_{s2,t2}^{\left(k\right)}$ is normally distributed around the validity level from the previous trial $x_{s2,t2}^{\left(k-1\right)}$, with the variance of this distribution depending on the inferred volatility $x_{s3,t3}^{\left(k\right)}$. Here, the free parameter κ describes how strongly the estimated volatility will influence validity level learning, and ω is a constant component of the learning step size. Finally, at the highest level (Eq 3), the inferred volatility x_s3,t3_ is normally distributed around the inferred volatility from the previous trial, with the variance of this distribution (i.e., the speed of learning about the volatility) described by a free parameter *ϑ*.

During the fitting of the model to the data, one can estimate the trial-by-trial time-series (at each level *i*) of the participants’ beliefs $\mu_{i}^{\left(k\right)}$(i.e., posterior means of states $x_{i}^{\left(k\right)}$) and the updates on these beliefs $\varepsilon_{i}^{\left(k\right)}\;$(precision-weighted PEs) after observing a target. The variational approximation in the HGF provides analytic update equations describing these time-series:

$$\mu_{i}^{\left(k+1\right)}\;-\;\mu_{i}^{\left(k\right)}\;\sim \;\psi_{i}^{\left(k\right)}\delta_{i-1}^{\left(k\right)}\;=\;\varepsilon_{i}^{\left(k\right)},$$ (4)

$$\psi_{i}^{\left(k\right)}\;=\;\frac{{\hat{\pi }}_{i-1}^{\left(k\right)}}{\pi_{i}^{\left(k\right)}},$$ (5)

$$\pi_{i}^{\left(k\right)}\;=\;\frac{1}{\sigma_{i}^{\left(k\right)}}.$$ (6)

As shown in Eqs 4–6, in each trial, a belief update $\mu_{i}^{\left(k+1\right)}\;-\;\mu_{i}^{\left(k\right)}$ is proportional to the PE at the level below $\delta_{i-1}^{\left(k\right)}$, weighted by a precision ratio $\psi_{i}^{\left(k\right)}$. This precision ratio depends on the precision (inverse variance) of the prediction at the level below ${\hat{\pi }}_{i-1}^{\left(k\right)}$ and at the current level $\pi_{i}^{\left(k\right)}$. The superscript ^ denotes “prediction”: ${\hat{\mu }}_{1}^{\left(k\right)}$ is the prediction on trial k before observing the trial outcome, and ${\hat{\pi }}_{i}^{\left(k\right)}$ is the precision of this prediction. After applying these update equations to specific hierarchical levels, we obtain:

$$\mu_{3}^{\left(k+1\right)}-\mu_{3}^{\left(k\right)}\;\sim \;\psi_{3}^{\left(k\right)}\delta_{2}^{\left(k\right)}\;=\;\varepsilon_{3}^{\left(k\right)},$$ (7)

$$\delta_{2}^{\left(k\right)}\;=\;\frac{\sigma_{2}^{\left(k\right)}+{\left(\mu_{2}^{\left(k\right)}-\mu_{2}^{\left(k-1\right)}\right)}^{2}}{\sigma_{2}^{\left(k-1\right)}+e^{{\kappa \mu }_{3}^{\left(k-1\right)}+\omega }}-1,$$ (8)

$$\mu_{2}^{\left(k+1\right)}-\mu_{2}^{\left(k\right)}\;\sim \;\psi_{2}^{\left(k\right)}\delta_{1}^{\left(k\right)}\;=\;\varepsilon_{2}^{\left(k\right)},$$ (9)

$$\delta_{1}^{\left(k\right)}\;=\;\mu_{1}^{\left(k\right)}-{\hat{\mu }}_{1}^{\left(k\right)},$$ (10)

$${\hat{\mu }}_{1}^{\left(k\right)}\;=\;s(\mu_{2}^{\left(k-1\right)}).$$ (11)

At the lower level, the PE about the observed target $\delta_{1}^{\left(k\right)}$ is simply the difference between the actual and the predicted target (Eq 10), in which the prediction is a sigmoid function of the previous trial’s prediction about the validity level (Eq 11). This PE, weighted by the corresponding precision ratio, is used to update the predictions about the validity level in the next trial (Eq 9). At the higher level, the PE about the validity level (Eq 8; cf. [23] for a detailed explanation) is used to update the prediction of volatility in the next trial (Eq 7). These HGF-derived time-series (specifically, |${\hat{\mu }}_{2}^{\left(k\right)}|$ and ${|\varepsilon }_{2}^{\left(k\right)}|$)–fitted to each participant’s behavioural data—were used as regressors in subsequent analysis of MEG data. Prior variance $log\;(\sigma_{2}^{\left(0\right)})$ was treated as a free parameter.

Finally, to map the agent’s beliefs onto the observed behavioural data, we specified a response model for categorical outcomes (a binary softmax function of the agent’s predictions), where the probability of a particular outcome *y =* {0; 1} is described by the logistic sigmoid:

$$p(y|{\hat{\mu }}_{1},\zeta )\;=\;s(-\zeta (2{\hat{\mu }}_{1}-1)(2y-1)).$$ (12)

The free parameter *ζ* encodes the decision noise. Here, because we had 2 time-series of predictions corresponding to the target location and latency (${\hat{\mu }}_{1s}$ and ${\hat{\mu }}_{1t}$), and they could be either relevant (e.g., ${\hat{\mu }}_{1s}$ in trials corresponding to the spatial task) or irrelevant (e.g., ${\hat{\mu }}_{1s}$ in trials corresponding to the temporal task), we parameterized the response model such that both relevant and irrelevant predictions could explain behaviour with different weights:

$$p(y_{s,t}|{\hat{\mu }}_{1s,t},\zeta )\;=\;s(-\zeta_{s,t}^{rel}(2{\hat{\mu }}_{1s,t}-1)(2y_{s,t}-1)-\zeta_{s,t}^{irrel}(2{\hat{\mu }}_{1t,s}-1)(2y_{s,t}-1)).$$ (13)

Thus, in a spatial task, both spatial predictions ${\hat{\mu }}_{1s}$ (via $\zeta_{s}^{rel}$) and temporal predictions ${\hat{\mu }}_{1t}$ (via $\zeta_{t}^{irrel}$) may have been used to model the observed response. Responses *y* were coded in contingency space, thus the mapping of y onto its possible values {0; 1} was identical to the mapping of x_s1,t1_ onto {0; 1}.

To select a model that best describes our observed data, we designed 5 alternative observation models (HGF3s with context-specific or nonspecific learning parameters ω; HGF2s, where changes in volatility are not inferred as κ = 0, again with context-specific or nonspecific learning parameters ω; and a standard reinforcement-learning model with a fixed learning rate) and 2 response models (Eq 13) in a factorial manner. Thus, our HGF observation model could consist of 3 levels (with a free parameter κ) or 2 levels (with κ = 0). As a result, in the reduced HGF2, the volatility estimates were decoupled from the lower levels and did not influence behaviour. Furthermore, the HGF learning parameters ω were either context-specific (i.e., with free parameters *ω*^*rel*^ and *ω*^*irrel*^ estimated for relevant and irrelevant contexts respectively, see Eqs 14 and 15 below) or nonspecific (whereby a single free parameter ω was estimated for both contexts, as in Eq 8).

$$\{\begin{array}{l} \delta_{2s}^{\left(k\right)}\;=\;\frac{\sigma_{2s}^{\left(k\right)}+{\left(\mu_{2s}^{\left(k\right)}-\mu_{2s}^{\left(k-1\right)}\right)}^{2}}{\sigma_{2s}^{\left(k-1\right)}+e^{\kappa \mu_{3s}^{\left(k-1\right)}+\omega_{s}^{rel}}}-1\;if\;spatial\;task\\ \delta_{2s}^{\left(k\right)}\;=\;\frac{\sigma_{2s}^{\left(k\right)}+{\left(\mu_{2s}^{\left(k\right)}-\mu_{2s}^{\left(k-1\right)}\right)}^{2}}{\sigma_{2s}^{\left(k-1\right)}+e^{\kappa \mu_{3s}^{\left(k-1\right)}+\omega_{s}^{irrel}}}-1\;if\;temporal\;task; \end{array}$$ (14)

$$\{\begin{array}{l} \delta_{2t}^{\left(k\right)}\;=\;\frac{\sigma_{2t}^{\left(k\right)}+{\left(\mu_{2t}^{\left(k\right)}-\mu_{2t}^{\left(k-1\right)}\right)}^{2}}{\sigma_{2t}^{\left(k-1\right)}+e^{\kappa \mu_{3t}^{\left(k-1\right)}+\omega_{t}^{rel}}}-1\;if\;temporal\;task\\ \delta_{2t}^{\left(k\right)}\;=\;\frac{\sigma_{2t}^{\left(k\right)}+{\left(\mu_{2t}^{\left(k\right)}-\mu_{2t}^{\left(k-1\right)}\right)}^{2}}{\sigma_{2t}^{\left(k-1\right)}+e^{\kappa \mu_{3t}^{\left(k-1\right)}+\omega_{t}^{irrel}}}-1\;if\;spatial\;task. \end{array}$$ (15)

Similarly, our response model could include both the relevant and irrelevant predictions (with free parameters *ζ*^*rel*^ and *ζ*^*irrel*^), or only the relevant predictions (with *ζ*^*irrel*^ = 0). Additionally, as an alternative observation model not based on the HGF, we considered a standard reinforcement learning based on the Rescorla-Wagner formulation [65], with 2 free parameters representing fixed learning rates of location and latency, respectively. Models were compared using their free-energy approximation to log-model evidence in a random-effects Bayesian model selection procedure [32]. The prior and posterior means ± SD for all free parameters of the winning model are shown in Table 3.

**Table 3:** Optimised parameters of the winning HGF model.

Parameter	Description	Prior Mean	Prior SD	Posterior Mean	Posterior SD
$log\;(\sigma_{2s}^{\left(0\right)})$	Prior variance of spatial predictions	0.10	4	0.10	0.01
$log\;(\sigma_{2t}^{\left(0\right)})$	Prior variance of temporal predictions	0.10	4	0.09	0.02
$\omega_{s}^{\left(rel\right)}$	Learning rate: location, spatial task	−5	1	−5.13	0.15
$\omega_{t}^{\left(rel\right)}$	Learning rate: latency, temporal task	−5	1	−5.11	0.20
$\omega_{s}^{\left(irrel\right)}$	Learning rate: location, temporal task	−5	1	−5.17	0.23
$\omega_{t}^{\left(irrel\right)}$	Learning rate: latency, spatial task	−5	1	−5.22	0.53
$log\;(\zeta_{s}^{\left(rel\right)})$	Decision noise: location, spatial task	1.38	1	0.64	0.14
${log\;(\zeta }_{t}^{\left(rel\right)})$	Decision noise: latency, temporal task	1.38	1	0.67	0.18

### MEG acquisition and analysis

MEG data were acquired using a 275-channel whole-head setup with third-order gradiometers (CTF MEG International Services LP, Coquitlam, British Columbia, Canada) at a sampling rate of 1200 Hz. Eye movements and pupil size data were recorded using a nonferrous infrared eye-tracking system (SR Research, Ottawa, Ontario, Canada). All subsequent analyses were performed in SPM12 (Wellcome Trust Centre for Neuroimaging, University College London), except where noted.

Continuous data were high-pass filtered at 0.1 Hz and notch-filtered at 50 Hz to remove slow drifts and line artefacts, and downsampled to 300 Hz. The vertical eye-tracker data were used to detect blinks. Sensor data were corrected for blink artifacts by subtracting their 2 principal modes [66]. To reduce the dimensionality of the data for subsequent analysis, we identified the main sources involved in processing task stimuli using multiple sparse priors under group constraints [67]. Here, artefact-corrected data were epoched between −1000 and 1000 ms relative to cues and targets, low-pass filtered at 48 Hz and baseline-corrected relative to the last 100 ms before cue or target onset by subtracting the average of the baseline period. Epoched data (960 trials per condition) were averaged per channel and condition using robust averaging [68]. Per participant, we calculated 3D source activity maps corresponding to the evoked activity in the 0–400 ms (Hanning) window relative to cue and target onset as well as their respective baselines (−400 to 0 ms). The primary sources involved in cue (target) processing were identified as clusters of significant differences between postcue (post-target) and precue (pretarget) source activity maps using GLMs with factors participant and epoch (post versus pre), after thresholding and correcting the statistical parametric maps at a peak-level *p*_FWE_ < 0.05. Additionally, to identify sources involved in differential processing of cues and targets, we calculated 3D source activity maps of total (evoked and induced) activity (0–400 ms relative to cue and target) present in the data after band-pass filtering the entire epochs between 1 and 48 Hz, and contrasted the ensuing activity maps related to cue- versus target-processing in a GLM with factors participant, stimulus (cue versus target), and epoch (post versus pre), thresholding and correcting the statistical parametric maps at a peak-level *p*_FWE_ <0.05. Sources were labeled using the SPM12 atlas provided by Neuromorphometrics, Inc. Significant clusters were then used to extract individual participants’ source-level time-series using a linearly constrained minimum variance beamformer [69], as implemented in the Data Analysis in Source Space (DAiSS) toolbox for SPM12 (https://github.com/SPM/DAiSS). Source-level time series, extracted from continuous data after high-pass and notch filtering, but before the remaining preprocessing steps, were transformed into a TF representation (frequency range: 8–48 Hz, frequency step: 2.5 Hz, frequency smoothing: ±2 Hz) using a sliding Hanning tapered window (length: 400 ms, time step: 20 ms) as implemented in the mtmconvol function of the FieldTrip toolbox (http://www.fieldtriptoolbox.org/). TF data were log-transformed, averaged per source across hemispheres, and entered into a convolution analysis for TF responses [34].

#### Convolution modelling

**Fig 5:**
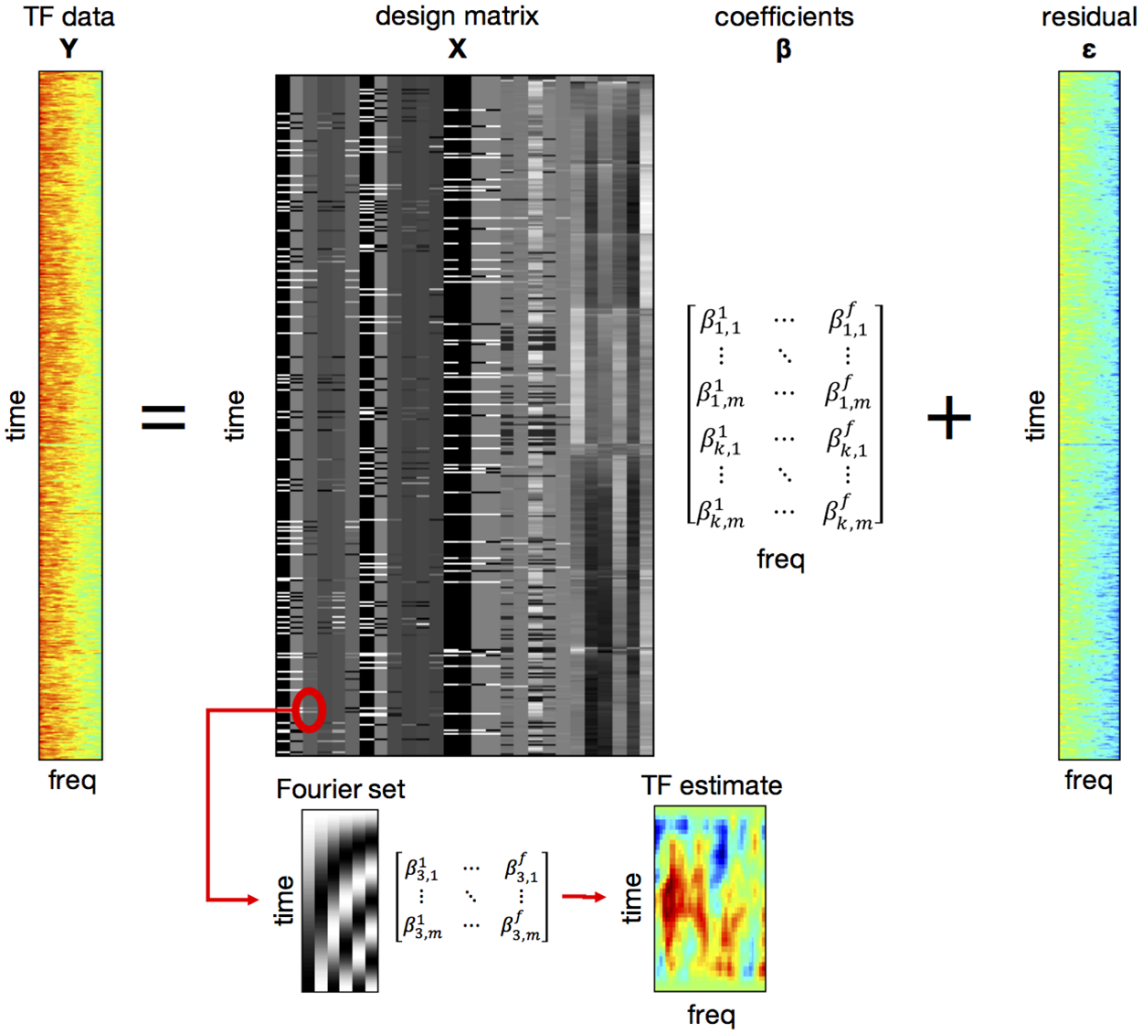
Convolution modelling for TF responses. TF data from the entire experiment (without epoching) were modelled using a GLM approach, with the design matrix specifying event and nuisance regressors (columns, left to right: cue onset and its 5 modulation regressors; target onset and its 5 modulation regressors; response onset and its 3 modulation regressors; 5 EOG and pupil size nuisance regressors; 6 motion regressors; see main text for details). Because each regressor was modelled as a Fourier time series (inset below), the resulting Fourier coefficients (here depicted for the third out of k columns and corresponding to parameter estimates for m basis functions and f frequencies) constitute a deconvolved TF response to each event type and/or parametric regressor. Data pertaining this figure are available on Figshare https://figshare.com/s/2d2755bfdeea1cbb415f. GLM, general linear model; EOG, electrooculography; freq, frequency; TF, time-frequency.

Rather than epoching the data, we modelled the continuous TF data (low-pass filtered at 300 Hz) estimated for the entire session using several regressors (Fig 5). Convolution modelling ([34]; see [70] for an application) is formally equivalent to testing for the effects of trial-specific explanatory variables at each point in peristimulus time but allows for overlapping responses to successive trials (in the same way that fMRI timeseries are modeled). The regressors included experimental regressors coding for cue, target, and response onsets as well as nuisance regressors. Blinks were detected in the vertical eye-tracker channel by detecting time points for which the temporal derivative of the vertical eye-tracker exceeded its mean + 3 SDs. Pupil size data were corrected for blinks by interpolating the data points from −15 ms to 385 ms relative to blink onset. Corrected pupil size time-series, as well as horizontal and vertical eye-tracker time-series and their temporal derivatives were used as nuisance regressors in the convolution GLMs. Furthermore, based on continuous head movement measurement inside of the MEG scanner, we calculated 6 movement parameters (3 translations and 3 rotations; cf. [71]), which were used as further nuisance regressors.

The event regressors coded for cue, target (each containing 960 entries), and response onsets (960 minus the number of trials with no responses). The cue onset regressor was additionally modulated by parametric regressors coding for task (spatial versus temporal) and the HGF-based inferred predictions about cue validity $\left|{\hat{\mu }}_{2}^{\left(k\right)}\right|$ (separately for spatial relevant, spatial irrelevant, temporal relevant, and temporal irrelevant predictions). Similarly, the target onset regressor was modulated by parametric regressors coding for task and the HGF-based inferred precision-weighted PEs about target location (latency) $\left|\varepsilon_{2st}^{\left(k\right)}\right|$, separately for the spatial/temporal and relevant/irrelevant contexts (as above). Finally, the response onset regressor was modulated by parametric regressors coding for response speed (1/RT), button, and outcome (correct or incorrect). The HGF-derived based regressors were largely uncorrelated (all r < 0.25; see Results and Fig 2D).

We modelled total power (encompassing induced and evoked responses) with a time range of −250 ms to 500 ms relative to events of interests (cue and target onsets), and a frequency range of 8–48 Hz. Each event regressor (i.e., coding for experimental events and their parametric modulations) was convolved with a sixth-order Fourier basis set, allowing for an estimation of TF responses (or their regressor coefficients) with the time-courses of 8–48 Hz power estimates modulated up to 8 Hz (750 ms epoch length at the highest-order Fourier basis function). The resulting TF maps of regressor coefficients were converted into 2D images and entered into second-level GLMs.

The group-level effects of predictions $\left|{\hat{\mu }}_{2}{}^{\left(\text{k}\right)}\right|$ and precision-weighted PEs |ε_2st_^(k)^| were inferred per cortical source by entering the single-participant regressor coefficient maps into 2 GLMs with factors participant, task (spatial, temporal), and context (relevant, irrelevant). To correct for multiple comparisons across cortical sources and regressors, the second-level statistical parametric maps were subject to F-tests across conditions at a peak-level threshold *p* < 0.05, Bonferroni-corrected across the investigated brain regions. To correct for multiple comparisons across TF points, we used a cluster-level family-wise error correction at *p* < 0.05 under random field theory assumptions [72]. In an exploratory analysis of TF responses in a higher frequency range (52–90 Hz, with all remaining analysis settings identical to the main reported analysis), no additional significant clusters of relevance, task, and/or interaction effects were observed.

#### DCM

To characterize the effective connectivity mediating the observed effects of prediction and PE signaling (and their modulation by contextual relevance) on oscillatory activity, we used DCM for TF responses ([35]; cf. [73] for an example application)—a phenomenological model that quantifies effective (directional) connectivity between different regions and frequency bands, without making assumptions about the underlying neurophysiological mechanisms mediating this connectivity (as is the case in e.g., DCM for evoked responses or stationary cross-spectra; the latter was not used here given the nonstationarity of the observed effects over time). DCM was used to quantify the within- and cross-frequency amplitude–amplitude coupling in 2 time windows: 0–500 ms relative to the cue onset, and 0–500 ms relative to the target onset. In the analysis of the neural responses to the cue (which were modulated by predictions), connectivity was modelled in a network of 4 sources identified in the analysis of cue-induced prediction signalling and its modulation by relevance: A1, MTG, TPJ, and V1. Similarly, when analysing target-induced activity (modulated by PEs), connectivity was modelled in a network of 2 sources: V1 and TPJ.

In each case, the TF maps of the parametric effects of predictions (respectively PEs), as well as their modulation by relevance and task, were averaged across participants. A “full” DCM model was designed, containing reciprocal connections between all regions and intrinsic (self-)connections in each region, all of which could be modulated by relevance and task. All of these modulatory effects were set to be nonlinear, i.e., allowed for amplitude-amplitude coupling between different frequency bands. The full model was fitted to the observed grand-average data and optimised using Bayesian model reduction [74], whereby “reduced” models—in which different subsets of parameters (connections) were fixed to 0 and not allowed to be modulated by relevance and/or task—were scored for their model evidence. This application of Bayesian model reduction provides a Bayesian model average across all reduced models, in which the contribution of each reduced model’s parameters to the average is weighted by this model’s evidence. Modulatory connectivity parameters describing the effect of contextual relevance on “baseline” prediction (or PE) signalling were considered significant when their posterior probability exceeded 99.9%.

## Abbreviations

A1: primary auditory cortex
DAiSS: Data Analysis in Source Space
DCM: dynamic causal modelling
GLM: general linear model
HGF: Hierarchical Gaussian Filter
HGF2: 2-level HGF
HGF3: 3-level HGF
MEG: magnetoencephalography
MTG: middle temporal gyrus
PE: prediction error
SMG: supramarginal gyrus
TF: time-frequency
TPJ: temporoparietal junction
